# A psychometric evaluation of the NICHD Parent-Infant Interaction Scales to inform clinical practice

**DOI:** 10.3389/fpsyg.2026.1773282

**Published:** 2026-03-03

**Authors:** Kyla Vaillancourt, Kim Alyousefi-van Dijk, Jane Barlow, Lydia Barge, Harshita Kavia, Camilla Rosan, Helen Sharp, Susan Ayers

**Affiliations:** 1 School of Health and Medical Sciences, City St George’s University of London, London, United Kingdom; 2 Anna Freud Centre, London, United Kingdom; 3 Department of Clinical, Educational and Health Psychology, University College London, London, United Kingdom; 4 Department of Social Policy and Intervention, University of Oxford, Oxford, United Kingdom; 5 Department of Primary Care and Mental Health, University of Liverpool, Liverpool, United Kingdom

**Keywords:** parent-infant interaction, parental sensitivity, reliability, validity, psychometrics, perinatal mental health

## Abstract

**Background:**

There is no recommended measure of parent-infant interaction that is psychometrically robust, feasible (i.e., brief and simple to use) and validated for use from birth to 12 months for routine use in Perinatal Mental Health Services (PMHS). This study tested the cross-sectional construct validity of the global sensitivity scale and a sensitivity composite from the NICHD Parent-Infant Interaction scales in a clinical sample of parents and babies, and the inter-rater reliability of all the NICHD scales in a sub-sample of dyads with infants under 3 months of age.

**Methods:**

Secondary analysis using parent-infant interaction videos from a Randomized Controlled Trial in specialist PMHS in England were used. Participants were 275 dyads who completed baseline self-reports (parental mental health symptoms, parent-reported bonding) and parent-infant observation tasks where sensitivity was measured (free play, book sharing, clothing change). Parents with infants over 2 months of age (*N* = 180), also completed measures of child development. Non-parametric correlations and linear regression were conducted to assess construct validity and intra-class correlations were conducted to evaluate inter-rater reliability.

**Results:**

Amongst dyads with infants 0–3 months, inter-rater reliability was good for the global and composite sensitivity scale, but poor-to-moderate for the scales of parental intrusiveness, dyadic mutuality and the infant scales. In the full sample of dyads, there was a small but significant negative association between the global and composite sensitivity scales and parental mental health symptom severity, but this association was not statistically significant when sensitivity was observed in the free play alone. In terms of child development, greater sensitivity was only associated with fewer socio-emotional problems when it was observed during the clothing change task. There was a statistically significant negative association between observed sensitivity and bonding difficulties, and the strength of this association was greater for younger infants than older infants.

**Conclusion:**

These findings contribute to the evidence base of the NICHD scales in a PMHS setting and suggest ways that the clinical utility of the NICHD scales could be improved for routine practice.

## 1 Introduction

Parental sensitivity—noticing, interpreting, and responding appropriately to infant cues—is one of the key mechanisms through which perinatal mental health problems can impact on children’s development (Aktar et al., 2019). Parents with clinical diagnoses of depression, personality disorder, or severe mental illness have been observed to be less sensitive in interactions with their infants (Biaggi et al., 2024; Bind et al., 2021; Murray et al., 1996; Vilaseca et al., 2025; Wendland et al., 2023), and some difficulties have been found for parents with PTSD or anxiety although findings are more mixed (Challacombe et al., 2016; Cook et al., 2018; Ierardi et al., 2019; Murray et al., 2007). Of all perinatal mental health problems, maternal depression has been most studied. A meta-analysis of 48 studies found a small but significant effect size (*r* = -0.16) between maternal depression and maternal sensitivity (Bernard et al., 2018). There is also evidence from other meta-analyses that there is a small but significant relationship between parental sensitivity and children’s internalizing and externalizing problems (*r* = –0.08 to –0.14) (Borairi et al., 2024; Cooke et al., 2022) and parental sensitivity and children’s language and cognitive outcomes (*r* = 0.25 –0.27) (Madigan et al., 2019; Valcan et al., 2018). Borairi et al. (2024) have also reported a small but significant indirect pathway of parental sensitivity as a mediator between maternal depression and both internalizing and externalizing problems in children, suggesting that parental sensitivity is one modifiable factor that may improve outcomes for children where a parent is experiencing depression.

Clinical services require effective ways of identifying parent-infant dyads most in need of intervention, as well as ways to evaluate treatments that are offered (whether that be for the parent or the dyad). Currently, there is no consensus on the best observational measure of parent-infant interaction to use to meet these aims (Marriott et al., 2019). In the UK, few clinical services use an observational measure in a formal way, and the desire for brief, easy to use measurement tools is often cited as a reason for using parental self-report measures in routine practice, rather than observational measures (Olander et al., 2021; Szaniecki and Barnes, 2016; Wright et al., 2023). For example, parent report measures of bonding (e.g., Postpartum Bonding Questionnaire or Mother Object Relations Scale; Royal College of Psychiatrists [RCPsych], 2018) may be used in PMHS and whilst these measures provide useful information about a parent’s perception of the bond, there is inconsistency in the literature about the strength of association between perceived bonding and the observed quality of parent-infant interaction (Muzik et al., 2017, 2013; Nath et al., 2020).

In 2018, the UK *Framework for Routine Outcomes Measures (FROM) in Perinatal Psychiatry* (Royal College of Psychiatrists [RCPsych], 2018) recommended three potential observational measures of parent-infant interaction for use in PMHS: the National Institute of Child Health and Development (NICHD) Scales (Cox and Crinic, 2006), the Parent-Infant Interaction Observation Scales (PIIOS) (Svanberg et al., 2013) and the Child–Adult Relationship Experimental (CARE) Index (Crittenden, 2001). Two systematic reviews of the psychometric properties for observational measures of parent-infant interaction have concluded that despite there being many available measures, most lack robust evidence (Lotzin et al., 2015; Shone et al., 2025). Only two of the three measures (i.e., CARE Index, PIIOS) recommended in the FROM were included in these reviews with evidence in at least one psychometric domain. The NICHD scales were not included in either systematic review, although studies examining the psychometric properties of the NICHD scales in community samples do exist with evidence of reliability, construct validity, and predictive validity (e.g., Forrer et al., 2024a; Nordahl et al., 2020; Sharp et al., 2024). Thus, while there is some evidence regarding the psychometric properties of these selected measures, evaluations have generally not been conducted with clinical populations.

An additional challenge for services is the clinical utility of these measures; in particular the time intensive training and administration associated with their use (Marriott et al., 2019). Sharp et al. (2024) aimed to address challenges with clinical utility by evaluating both the PIIOS and NICHD Scales, testing the reliability and predictive validity of different lengths of observations (3, 5, and 7 minutes). They found that a 5 minutes observation provided good inter-rater reliability and predicted later child socio-emotional problems for both measures. They also found that 3 scales from the NICHD Scales (global sensitivity, positive regard and reversely scored intrusiveness; a 3-scale composite) performed best of all tested options. In contrast, the PIIOS was only valid when all 13 items were included. Thus, findings from this study suggest that the NICHD Scales could be used in a shortened way to improve clinical utility. The potential to shorten the NICHD Scales also aligns with recent work that has tested a practitioner-version of the NICHD Scales that is derived from the global sensitivity scale (Forrer et al., 2024b).

When considering characteristics that are required for observational assessment in clinical practice there is an emphasis on brevity, simplicity and scalability (Forrer et al., 2024b). However, there are many observational assessment tools where the emphasis is on richness and comprehensiveness, such as multidimensional profiles of relationship quality (e.g., What to Look For in Relationships Scale, Osofsky et al., 2023) or patterns of synchrony/contingency (Leclère et al., 2014). Research that contributes to the evidence-base for assessments with different aims and purposes is needed to advance research and practice.

### 1.1 This study

In the present study, the aim is to test the psychometric properties of a specific construct from the NICHD scales to inform approaches that may improve feasibility for routine use in PMHS. The focus of this investigation is the parental sensitivity construct from the NICHD scales and will build on existing research which has aimed to improve the clinical utility of this system (Forrer et al., 2024b; Sharp et al., 2024). In particular this study will test if shortened versions of the NICHD Scales (the global sensitivity scale and a 3-scale sensitivity composite; Sharp et al., 2024) are valid in a clinical sample of parents (experiencing both perinatal mental health conditions and bonding difficulties), and their infants, by evaluating the cross-sectional associations with parental mental health symptoms, parent-reported bonding difficulties and parent-reported cognitive, language and socio-emotional child development. The validity of the parent scales was the focus of this investigation due to the potential for a sensitivity scale (or composite) to guide commonly delivered treatments in services such as video-feedback interventions that have parental sensitivity as a focus (O’Hara et al., 2019).

One aspect of construct validity as defined by the Consensus based Standards for the selection of health status Measurement Instruments (COSMIN) refers to the degree to which scores of an instrument are consistent with hypotheses (e.g., demonstrate relationships to scores of other instruments) (Mokkink et al., 2019). In addition, and to inform clinical decisions about the context for observing parent-infant interactions, this study also examined the construct validity of parental sensitivity measured in different observation tasks. There is evidence from the National Institute for Child Health and Development Study of Early Child Care and Youth Development (NICHD-SECYYD) dataset that structured observation tasks at age 2 years elicit more directive parenting behavior than unstructured free-play tasks, and parenting observed during structured tasks has been shown to be more predictive of child outcomes at age 4 years than parenting observed in unstructured tasks (Nordahl et al., 2020). We are not aware of any research that has examined how parenting observed in different observation tasks in the first year postpartum relate to parental factors or child outcomes earlier in development.

A further question is if the NICHD Scales can be applied reliably for dyads with infants younger than 3 months old because they were originally developed for use with children from 3 months of age. This is pertinent for UK Perinatal Mental Health Services (PMHS) that support parents with infants that range from newborn up to age two. There is pre-existing evidence regarding the inter-rater reliability of the NICHD scales in infants older than 3 months (Sharp et al., 2024), however we are only aware of one study that has tested the reliability of the NICHD scales with younger infants. Lakes et al. (2017) reported good inter-rater reliability for all of the NICHD scales in their sample from a neonatal unit but note that there were many cases where there was “no opportunity to observe” what was required to rate the infant scales.

Based on previous research, it was hypothesized that there would be a small but significant association between greater sensitivity (measured using the global sensitivity scale and the 3-scale sensitivity composite) and less severe maternal mental health symptoms and better child development outcomes. These associations were expected for sensitivity observed overall and in each observation task. No specific hypothesis was made about the strength of association between self-reported bonding and observed sensitivity. With regards to inter-rater reliability, we expected that better inter-rater reliability would be obtained for the parent scales than the infant scales in dyads with infants under 3 months of age.

## 2 Materials and methods

### 2.1 Study design

The study involves secondary analysis of data from a randomized controlled trial (RCT) evaluating the effectiveness of the Circle of Security-Parenting (COS-P) Programme in specialist PMHS in England (Rosan et al., 2023, ISRCTN18308962). PMHS are secondary care mental health services for women and birthing people who have moderate-severe or complex mental health needs in pregnancy or the within the first 2 years postpartum, offering multi-disciplinary care and treatment (Royal College of Psychiatrists [RCPsych], 2021). The inclusion criteria for parents to participate in the RCT were: birthing parents accessing care from one of ten participating PMHS, 18 years or older, able to attend a parenting group without being under the influence of substances, not experiencing active psychosis and having a child aged 0–12 months with no severe illness or developmental disorder. Eligible participants also had to have an average score of 1.1 or more on the Clinical Outcomes in Routine Evaluation-10 (CORE-10) or 1.0 or more on the CORE- Outcome Measure (CORE-OM) and 12 or more on the total score for the Postpartum Bonding Questionnaire (PBQ). Further information about the trial and the CONSORT diagram is described in Rosan et al. (2025). Assessments were conducted at baseline, and at 3, 7 and 12 months after baseline. The present analysis uses baseline data only.

### 2.2 Measures

#### 2.2.1 The NICHD coding system

The quality of parent-infant interaction was measured using the revised manual for the Qualitative Ratings for Parent-Child Interaction (Cox and Crinic, 2006), which uses a 5-point global rating scale adapted from the 4-point NICHD-SECYYD system (Owen, 1992). The 5-point manual is designed for babies aged 3–15 months of age but in this study was applied to parents and their infants ranging from newborn to 12 months of age. Parent-infant interactions were recorded online for approximately 10 min with parents based in their home, including one unstructured task (free play with no toys) and two structured tasks (book sharing and a clothing change). In line with other RCTs conducted with perinatal populations (Stein et al., 2018) a variety of observation tasks were included to elicit a range of infant and parent behaviors. This included a task that was likely to cause mild distress for the infant (clothing change task) to provide opportunities for parental responses to distress to be observed. Each task was filmed between 2 and 3 min (see Supplementary Table 2).

The NICHD-SECYYD system includes nine parental scales, four infant scales and one dyadic scale (see Supplementary Table 1 for a description). Each dimension is rated using a 5-point scale from 1 (not at all characteristic), to 5 (highly characteristic). Where there was no opportunity to observe parental (e.g., sensitivity to distress) or infant behavior (e.g., positive or negative mood if infant was sleeping) raters assigned a numeric value for “code not code.” This category was included in the inter-rater reliability calculations.

For the inter-rater reliability analysis in dyads with younger infants, all NICHD scales were examined. For analyses pertaining to construct validity, the global sensitivity scale and a 3-scale sensitivity composite (Sharp et al., 2024) was used. Scores for the 3-scale sensitivity composite are a sum of global sensitivity, positive regard and intrusiveness (reverse scored) and range from 1 to 15. Internal consistency for the 3-scale sensitivity composite in the current study was α = 0.79 (across all tasks), α = 0.76 (free play), α = 0.77 (book sharing) and α = 0.75 (clothing change). Analyses that involved total observed sensitivity used the mean score derived from sensitivity ratings across the three interaction tasks.

#### 2.2.2 Clinical outcomes in routine evaluation—outcome measure

Parental mental health symptom severity was measured using the CORE-OM, a self-report measure designed to assess the effectiveness of psychological therapies. It is comprised of 34 items and items are rated on a scale from 0 (not at all) to 4 (most, or all of the time). The total score is the mean of all items and range from 0 to 4, with higher values indicating greater symptoms/poorer wellbeing. The total score has demonstrated good internal consistency, test-retest reliability, sensitivity to change and convergent validity with the Symptom Checklist-90-Revised (Evans et al., 2002).

#### 2.2.3 Postpartum Bonding Questionnaire

The PBQ is a parent-report measure of perceived bonding. It is comprised of 25 items with a 6-point Likert scale from “never” to “always.” Only the total score was used in this study. The total score has demonstrated high sensitivity to identify bonding disorders via clinical interview and good internal consistency (Brockington et al., 2006, 2001; Wittkowski et al., 2007). Total scores range from 0 to 125, with higher scores indicating more bonding difficulties.

#### 2.2.4 Ages and Stages Questionnaire Third Edition and Ages and Stages Questionnaire—Social-Emotional

The ASQ-3 and ASQ:SE were used as measures of child development. Three subscales from the ASQ-3 were selected in this analysis. Given prior research that has established positive associations between parental sensitivity and better cognitive, language and socio-emotional outcomes (Cooke et al., 2022; Madigan et al., 2019; Valcan et al., 2018), we chose to examine associations between sensitivity and all of the ASQ-3 subscales, except for the fine motor and gross motor subscales. The problem-solving subscale was selected to represent cognitive development, and the communication subscale was selected as a measure of language development. The personal-social subscale assesses if the child can achieve their self-help needs in an age-appropriate way; this subscale less clearly maps on to a single cognitive or language domain but will involve a range of developmental capacities, including cognitive, language and socio-emotional. Each subscale is made up of six items. Lower scores indicate poorer development.

The ASQ:SE is a broad measure of socio-emotional development covering areas such as self-regulation, compliance, social-communication, etc. It is made up of 19, 22 or 26 items, depending on the age version used, with higher scores indicating poorer socio-emotional development.

Both the ASQ-3 and ASQ:SE have been developed for children aged 2–60 months. Total scores were converted to z-scores using the sample mean and standard deviation (SD) to allow different age versions to be analyzed together. Procedures for missing items followed recommendations in the scoring manual. The ASQ-3 has demonstrated internal consistency, test-retest, inter-observer reliability and moderate-high agreement with the Battelle Developmental Inventory (Squires et al., 2009). The ASQ:SE has demonstrated good internal consistency and test-re-test reliability, average sensitivity and specificity with the Child Behavioral Checklist and Vineland Social-Emotional Early Childhood Scale was.82 –92, respectively (Squires et al., 2001).

#### 2.2.5 Demographic information

Parents provided information about their children’s age, protected characteristics and mental health conditions. Mental health conditions were self-reported. Ethnicity, socio-economic status, level of education, employment and income were extracted from the Client Service Receipt Inventory (CSRI) (Beecham and Knapp, 2001). Household income was dichotomized according to the median household income as defined by the UK Government (Department for Work and Pensions, 2025).

### 2.3 Participants

Participants were recruited from ten PMHS in England. A total of 386 parents consented to the trial. Of those, 275 consented to complete baseline parent-infant video observations and are included in the analysis. There was no difference between those who did and did not complete a parent-infant observation in terms of ethnicity or level of education. Parents with a household income below the median were more likely to complete a video than those above the median [76.9% vs. 67.7% vs., χ^2^(2) = 3.99, *p* = 0.05]. There was a trend for younger parents (24 years or younger) to be more likely to complete a video than older parents [χ^2^(2) = 3.43, *p* = 0.06]. Parents who did not complete a video had marginally higher CORE-OM scores [*M* = 2.05, vs. *M* = 1.90, *t*(345) = 1.89, *p* = 0.06]. There was no statistically significant difference between parents who did or did not complete a video in terms of total PBQ score. Due to problems with video quality, four videos could not be coded in their entirety and were excluded from the analysis. Valid observational data was as follows: free play (*n* = 272 dyads), book sharing (*n* = 270) and clothing change (*n* = 266).

Of those with valid observational data, 271 completed the CORE-OM, 270 completed the PBQ, 179 and 180 completed the ASQ3 and ASQ:SE, respectively. Those who did not complete an ASQ had infants that were significantly younger than those who did complete (mean 12 weeks vs. 26 weeks, *p <* 0.001). Most parents of infants under 3 months of age did not complete the ASQ-3 or ASQ:SE, in line with the age that these measures were designed for (i.e.,2 months or older). There was no statistically significant difference between those who did and did not complete the ASQ in terms of maternal age, maternal education, household income or CORE-OM scores.

### 2.4 Procedure

Prior to starting the intervention, participants completed baseline questionnaires and met online with a researcher to complete the mother-infant observations which were video-recorded. Interaction videos were coded by six trained coders, who had reached adequate reliability against a gold-standard rater on a set of training videos and were blind to treatment allocation. Each observation task was coded for a maximum of three and a half minutes. Coders additionally rated one video per month to check inter-rater reliability. ICCs for these videos (across all tasks) were 0.96 for global sensitivity, 0.97 for intrusiveness and 0.96 for positive regard, indicating excellent inter-rater reliability.

The original RCT received ethical approval from the Surrey NHS Research Ethics Committee on 26th of November 2021 (reference no: 21/LO/0723). No further ethical approval was required for the current study because it was a secondary analysis.

### 2.5 Analysis plan

Data was managed and analyzed using SPSS v. 29. Non-parametric Spearman’s correlations were conducted for analyses that used the PBQ and sensitivity due to non-normal distributions.

To test the relationship between sensitivity and child development, linear regression models were conducted, first to test simple associations and then adjusted for confounders (maternal age, maternal level of education, household income, mental health symptoms) if statistically significant associations were present in the simple associations. Confounding variables were chosen a priori based on previous literature. Hierarchical linear regression was used to examine the unique contribution of sensitivity in each observation task for children’s development. Demographics were first entered into the model, then maternal mental health symptoms and then sensitivity observed in each task was entered individually as separate steps. This stepwise model was repeated for each developmental domain. Variance Inflation Factors ranged from 1.0 to 1.8, indicating multicollinearity was within acceptable limits; therefore, all parenting variables were retained in the model. The assumption of normality of residuals was not met for all ASQ-3 subscales and thus bootstrapping using 2,000 resampling iterations was conducted. Bootstrapped coefficients and confidence intervals (CIs) are presented for all regression analyses.

To evaluate inter-rater reliability of the NICHD scales where infants were aged 0–3 months, intra-class correlation (ICCs) estimates, and their 95% confidence intervals, were calculated based on a mean-rating (*k* = 3), absolute-agreement, 2-way random-effects model. ICCs were interpreted as follows: poor reliability = 0.5, moderate reliability 0.5–0.75, good reliability 0.75–0.90 and excellent reliability = 0.90 (Koo and Li, 2016).

### 2.6 Sample size calculations

A minimum of 92 participants (5 predictors) or 103 participants (7 predictors) were required to detect a small effect size using a fixed linear multiple regression model, indicating sufficient sample size was available to test associations between parenting and child outcomes (Faul et al., 2009)

A sample size of 38 participants was used for the inter-rater reliability analysis, in order to achieve a minimally acceptable ICC of 0.6 (moderate) and a preferable ICC of 0.79 or higher, with 3 raters, two tailed 0.05 significance level and 80% power (Arifin, 2018).

## 3 Results

Descriptive statistics are presented first, followed by the inter-rater reliability of the NICHD scales in the sub-sample of young infants. Analyses relating to construct validity include the entire sample and are first presented for observed sensitivity (the global sensitivity scale and the 3-scale composite separately) across all tasks and then observed sensitivity individually for each task. Results that are not directly related to the primary results are included in Supplementary material and referenced in the text where appropriate.

### 3.1 Descriptives statistics

Table 1 describes the participant characteristics of those who completed parent-infant observations. Most participants identified as women but 2% of the sample described their gender identity as “non-binary” and therefore the term “parents” rather than “mother” is used throughout the manuscript to describe the sample. Approximately 13% of the sample were young parents (24 years or younger). Approximately 28% (*N* = 76) of infants were younger than 3 months old (i.e., 12 weeks) at the time of observation.

**TABLE 1:** Participant characteristics.

Variable	Mean (SD)	Minimum, maximum	N
Infant age (weeks)	21.10 (11.95)	3, 56	275
Maternal age (years)	30.70 (5.37)	18, 43	269
**N (%)**			
Infant sex (female)		138 (50.2)	275
Parent’s ethnicity	White British or Other White Background	262 (95.3)	275
	All other ethnic groups	13 (4.7)	
Parent’s gender identity	Woman	270 (98.2)	275
	Non-binary	5 (1.8)	
Parent’s sexual orientation	Bisexual	33 (12.0)	275
	Heterosexual	232 (84.4)	
	Lesbian	1 (0.4)	
	Other	3 (1.1)	
	Not known	6 (2.2)	
Parent’s relationship status	In a relationship	245 (89.1)	275
	In a relationship, not living together	9 (3.3)	
	Separated or single	21 (7.6)	
Parent’s highest level of education	Higher education	167 (60.7)	275
	Tertiary or further education	84 (30.5)	
	Secondary education	15 (5.5)	
	Primary education or less	1 (0.4)	
Household income	Weekly income > £373.00	146 (53%)	275
	Weekly income < = £373.00	129 (47%)	
Parent has other children	Yes	118 (42.9)	275
	No	153 (55.6)	
	Unknown	4 (1.5)	
Mental health condition (self-reported) ^a^	Depression	231 (84)	275
	OCD	30 (10.9)	
	Anxiety	234 (85.1)	
	Personality difficulties	37 (13.5)	
	Trauma	108 (39.3)	
	Psychosis	6 (2.2)	
	Bi-polar	11 (4.0)	
	Other	30 (10.9)	
	Unknown	2 (0.7)	
Prior MH condition (self-reported)	Yes	245 (90%)	275
	No	28 (10%)	

^a^Parents may have endorsed more than one mental health condition.

Descriptive statistics are presented for the CORE-OM, PBQ, ASQ-3, and ASQ:SE in Table 2. Infants were least likely to show distress during the free play task and most likely to show distress in the clothing change task. Statistics for observed parenting and intercorrelations are presented in Supplementary Tables 3, 4, respectively. Observed parenting was unrelated to maternal age, maternal ethnicity, maternal education, household income and infant sex.

**TABLE 2:** Descriptive statistics for parental mental health symptoms, parent-reported bonding, and children’s development outcomes.

Outcome	N		Mean	SD	Min, max
**Parent outcomes**					
CORE-OM^†^	271		1.90	0.59	.32, 3.4
PBQ^†^	270		34.27	16.43	3, 90
**Child outcomes**					
ASQ-3 (z-scores)	180	Communication	0.06	0.96	–3.14, 1.43
	180	Problem solving	0.06	0.97	–3.32, 1.07
	180	Personal social	0.06	0.98	–3.21, 1.23
ASQ:SE (z-scores)	181	Total score	–0.09	0.94	–1.75, 3.68

CORE-OM, Clinical Outcomes for Routine Evaluation-Outcome Measure, higher scores indicate greater symptoms; PBQ, Postpartum Bonding Questionnaire, higher scores indicate greater bonding difficulties; ASQ, Ages and Stages Questionnaire- Third Edition, lower scores indicate poorer development; ASQ:SE, Ages and Stages Questionnaire: Socio Emotional, higher scores indicate greater socio-emotional problems;

† CORE-OM and PBQ were re-administered at baseline; scores may therefore be lower than those recorded for eligibility screening.

### 3.2 Inter-rater reliability for all NICHD scales in very young infants

ICCs and 95% CIs are presented in Table 3 for the NICHD Scales for a random sample of 38 out of the total 76 dyads with infants under 3 months of age. A visual inspection of the main coder’s score distribution suggested that the selected cases reflected a broad range of parenting quality. ICCs were excellent or good for sensitivity to distress, sensitivity to non-distress, global sensitivity, positive regard, stimulation, animation and the 3-scale composite when parenting was observed across all tasks. Parental intrusiveness, dyadic mutuality and many of the infant scales demonstrated poor to moderate inter-rater reliability. CIs for some of the scales were extremely wide, indicating unreliable inter-rater reliability. The % unobservable (assigned a “could not code” rating) was also calculated for each scale based on ratings from the gold-standard rater. Apart from the sensitivity to distress scale which commonly has some cases that can’t be observed (i.e., if the infant is not distressed during the observation), it was largely the dyadic and infant scales where a proportion of cases (13–24%) could not be given a rating in the dyads with young infants.

**TABLE 3:** ICC and 95% CIs for the NICHD Parent-Infant Interaction Scales for dyads with infants under 3 months of age (*k* = 3, *n* = 38).

	Total	Free play		Book sharing		Clothing change	
Scale	ICC (95% CIs)	ICC (95% CIs)	% non-observable	ICC (95% CIs)	% non-observable	ICC (95% CIs)	% non-observable
Sensitivity to distress	0.93 (0.86, 0.96)	0.78 (0.63, 0.88)	52.6%	0.90 (0.83, 0.95)	55.3%	0.94 (0.89, 0.97)	48.6%
Sensitivity to non-distress	0.73 (0.42, 0.87)	0.53 (0.21, 0.73)	7.9%	0.66 (0.39, 0.82)	2.6%	0.69 (0.46, 0.83)	0%
Global sensitivity	0.81 (0.48, 0.92)	0.77 (0.56, 0.88)	0%	0.72 (0.40, 0.87)	0%	0.82 (0.64, 0.91)	0%
Intrusiveness	0.48 (0.12, 0.71)	0.53 (0.20, 0.74)	0%	0.56 (0.22, 0.77)	0%	0.29 (–0.33, 0.63)	0%
Detachment	0.59 (0.22, 0.79)	0.44 (0.01, 0.68)	0%	0.44 (0.08, 0.68)	0%	0.64 (0.38, 0.81)	0%
Stimulation	0.77 (0.41, 0.89)	0.79 (0.62, 0.89)	2.6%	0.79 (0.61, 0.88)	0%	0.64 (0.22, 0.83)	0%
Positive Regard	0.80 (0.60, 0.90)	0.79 (0.63, 0.88)	0%	0.63 (0.35, 0.79)	0%	0.76 (0.54, 0.87)	0%
Negative Regard	0.65 (0.36, 0.81)	0.54 (0.23, 0.75)	0%	0.21 (–0.20, 0.53)	0%	0.71 (0.50, 0.84)	0%
Animation	0.75 (0.29, 0.89)	0.81 (0.60, 0.90)	0%	0.67 (21, 0.85)	0%	0.64 (0.24, 0.82)	0%
Dyadic mutuality	0.50 (0.05, 0.74)	0.59 (0.20, 0.79)	13.2%	0.46 (0.10, 0.69)	18.4%	0.47 (0.05, 0.72)	21.6%
Positive mood	0.39 (0.02, 0.66)	0.53 (0.15, 0.74)	18.4%	0.37 (–0.01, 0.63)	18.4%	0.34 (–0.03, 0.61)	21.6%
Negative mood	0.60 (0.32, 0.78)	0.61 (0.34, 0.78)	13.2%	0.64 (0.38, 0.80)	18.4%	0.72 (0.52, 0.85)	16.2%
Activity level	0.62 (0.23, 0.81)	0.59 (0.21, 0.79)	15.8%	0.65 (0.40, 0.81)	18.4%	0.53 (0.20, 0.74)	16.2%
Sustained attention	0.70 (0.48, 0.84)	0.65 (0.40, 0.80)	18.4%	0.78 (0.60, 0.88)	18.4%	0.62 (0.36, 0.79)	24.3%
3-scale composite	0.76 (0.370, 0.90)	0.79 (0.56, 0.90)	0%	0.70 (0.30, 0.86)	0%	0.78 (0.55, 0.89)	0%

ICC = Intra Class Correlation; ICC: = 0–0.5 = poor reliability, 0.5–0.75 = moderate reliability, 0.75– 0.90 = good reliability, = 0.90 excellent reliability. % non-observable is based on ratings from gold-standard rater.

Since inter-rater reliability was good for the global sensitivity scale and the 3-scale composite, the subsequent analyses relating to construct validity represent all dyads including those with infants under 3 months of age. However, because of the poor inter-rater reliability for intrusiveness and this being one component of the 3-scale composite, results are presented in Supplementary material for younger and older infants separately (see Supplementary Table 5).

### 3.3 Construct validity

#### 3.3.1 Sensitivity observed across all tasks

There was a small but statistically significant negative association between global sensitivity and parental mental health symptoms (*r_*s*_* = –0.16, *p* = 0.01) and global sensitivity and parent-reported bonding difficulties (*r_*s*_* = –0.20, *p* = 0.001). The strength of association was the same for the 3-scale composite: parental mental health symptoms (*r_*s*_* = –0.16, *p* = 0.01), parent-reported bonding difficulties (*r_*s*_* = –0.20, *p* < 0.001).

There was no statistically significant association between global sensitivity and the ASQ-3 (communication, problem solving or personal social) or ASQ:SE (see Table 4). This was also the case for the 3-scale sensitivity composite.

**TABLE 4:** Estimates from bootstrapped linear regression of child development on total observed sensitivity, unadjusted.

	ASQ-3						ASQ:SE	
	Communication		Problem Solving		Personal Social		Socio-emotional	
Predictor	*B* (BCa 95% CI)	*p*	*B* (BCa 95% CI)	*p*	*B* (BCa 95% CI)	*p*	*B* (BCa 95% CI)	*p*
Global sensitivity	0.07 (-0.11, 0.25)	0.45	0.02 (-0.16, 0.20)	0.79	-0.06 (-0.23, 0.13)	0.52	-0.09 -0.26, 0.09)	0.35
3-scale composite	0.003 (-0.07, 0.08)	0.93	0.01 (-0.07, 0.08)	0.82	-0.02 (-0.10, 0.06)	0.58	-0.05 (-0.13, 0.03)	0.21

BCa CI, Bias corrected accelerated 95% bootstrap confidence interval (2,000 resampling iterations); ASQ-3, Ages and Stages Questionnaire- Third Edition, lower scores indicate poorer development; ASQ:SE, Ages and Stages Questionnaire: Socio Emotional, higher scores indicate greater socio-emotional problems.

The sensitivity analyses that were conducted by repeating the tests separately for younger and older infants were broadly consistent with what is reported above for the overall sample, except for parent-reported bonding where the strength of association with observed sensitivity was greater for younger infants (global sensitivity *r_*s*_* = –0.41, 3-scale composite *r_*s*_* = –0.40) than for older infants (global sensitivity *r_*s*_* = –0.10, 3-scale composite *r_*s*_* = –0.11) (see Supplementary Tables 5–7).

#### 3.3.2 Construct validity of sensitivity for each observation task

##### 3.3.2.1 Associations with maternal mental health and parent-reported bonding

There was a small but statistically significant negative association between global sensitivity and parental mental health symptoms when observed in the book sharing (*r_*s*_* = –0.19, *p* = 0.002) and clothing change (*r_*s*_* = –0.17, *p* = 0.01), but not the free play (*r_*s*_ = –*0.05). Similar findings were found for the 3-scale sensitivity composite free play: (*r_*s*_* = -0.09); book sharing (*r_*s*_* = –0.14, *p* = 0.03); clothing change (*r_*s*_* = –0.19, *p* = 0.002).

There was a small and statistically significant negative association between global sensitivity and bonding difficulties when observed in the book sharing (*r_*s*_* = -0.19, *p* = 0.001) and clothing change (*r* = -0.18, *p* = 0.01), but the negative association was not statistically significant in the free play (*r_*s*_* = -0.10). There was a small and statistically significant negative association between the 3-scale sensitivity composite in all three tasks and bonding difficulties: free play (*r_*s*_* = -0.13, *p* = 0.03); book sharing (*r_*s*_* = -0.15, *p* = 0.02), clothing change (*r_*s*_* = -0.22, *p* = 0.001).

##### 3.3.2.2 Associations with child development

There were no statistically significant associations between global sensitivity or the 3-scale sensitivity composite in each task and any of the ASQ-3 domains. For socio-emotional development, there was a negative and statistically significant association between sensitivity observed in the clothing change task and children’s socio-emotional development (see Table 5). When maternal age, maternal level of education, household income (Step 1), and maternal mental health symptoms (Step 2), were entered into the model followed by sensitivity in each task (Step 3: free play, Step 4: book sharing, Step 5: clothing change), the unique association between sensitivity measured in the clothing change task and socio-emotional development remained statistically significant for global sensitivity (*B* = -0.22). Greater household income and lower sensitivity observed during the clothing change task was associated with greater social-emotional difficulties (see Table 6). The final model accounted for 8% of the variance in ASQ:SE scores (*R*^2^ = 0.08), with a significant improvement in model fit compared to Step 4 (ΔF = 6.26, *p* < 0.05; ΔR^2^ = 0.03).

**TABLE 5:** Estimates from bootstrapped linear regression of child development on observed sensitivity by task, unadjusted.

	ASQ-3						ASQ:SE	
	Communication		Problem Solving		Personal Social		Socio-emotional	
Predictor	*B* (BCa 95% CI)	*p*	*B* (BCa 95% CI)	*p*	*B* (BCa 95% CI)	*p*	*B* (BCa 95% CI)	*p*
**Global sensitivity**								
Free play	0.10 (-0.07, 0.29)	0.25	0.12 (-0.09, 0.36)	0.24	-0.01 (-0.19, 0.19)	0.96	0.01 (-0.17, 0.20)	0.92
Book sharing	-0.10 (-0.29, 0.08)	0.32	-0.10 (-0.27, 0.06)	0.29	-0.12 (-0.29, 0.08)	0.26	0.09 (-0.08, 0.28)	0.31
Clothing Change	0.04 (-0.13, 0.23)	0.61	0.03 (-0.15, 0.20)	0.73	0.05 (-0.14, 0.24)	0.56	-0.17 (-0.34, -0.02)	0.03
**3-scale composite**								
Free play	0.04 (-0.04, 0.13)	0.30	0.06 (-0.03, 0.16)	0.17	0.01 (-0.07, 0.09)	0.79	-0.01 (-0.08, 0.07)	0.81
Book sharing	-0.05 (-0.12, 0.13)	0.23	-0.03 (-0.10, 0.04)	0.47	-0.05 (-0.13, 0.03)	0.20	0.06 (-0.02, 0.14)	0.15
Clothing Change	0.01 (-0.08, 0.09)	0.87	-0.01 (-0.09, 0.07)	0.90	0.02 (-0.07, 0.10)	0.66	-0.09 (-0.16, -0.01)	0.02

BCa CI, Bias corrected accelerated 95% bootstrap confidence interval (2,000 resampling iterations); ASQ-3, Ages and Stages Questionnaire- Third Edition, lower scores indicate poorer development; ASQ:SE, Ages and Stages Questionnaire: Socio Emotional, higher scores indicate greater socio-emotional problems.

**TABLE 6:** Estimates from a bootstrapped hierarchical linear regression of ASQ:SE on observed sensitivity by task, adjusting for demographic factors and maternal mental health symptoms.

	Global sensitivity				3-scale sensitivity composite			
Predictor	*B*	BCa 95% CI	β	*p*	*B*	BCa 95% CI	β	*p*
**Step 1**								
Maternal age^a^	0.03	-0.33, 0.41	0.01	0.89	-0.02	-0.40, 0.38	-0.01	0.93
Maternal education^b^	0.22	-0.07, 0.48	0.14	0.15	0.19	-0.10, 0.44	0.11	0.21
Household income^c^	-0.42	-0.72, 0.12	-0.22	0.01	-0.39	-0.70, -0.09	-0.21	0.01
**Step 2**								
Mental health symptoms^d^	0.23	-0.04, 0.53	0.14	0.12	0.19	-0.10, 0.50	0.12	0.17
**Step 3**								
Free play	0.02	-0.16, 0.19	0.02	0.84	-0.01	-0.08, 0.06	-0.01	0.88
**Step 4**								
Book sharing	0.15	-0.04, 0.37	0.16	0.13	0.07	-0.02, 0.17	0.17	0.14
**Step 5**								
Clothing change	-0.22	-0.39, 0 -0.06	-0.24	0.01	-0.09	-0.17, -0.02	-0.23	0.01

BCa CI, Bias corrected accelerated 95% bootstrap confidence interval (2,000 resampling iterations); Standardized beta coefficients (β) were based on the original sample and were not bootstrapped; ASQ:SE, Ages and Stages Questionnaire: Socio Emotional, greater scores indicate more socio-emotional problems;

^a^0 = older than 25 years, 1 = 24 years or younger;

^b^0 = higher education, 1 = tertiary education, 2 = secondary education or less;

^c^0 = not deprived (weekly income =£373.00), 1 = deprived (weekly income = £373.00;

^d^higher scores indicate greater symptoms.

Similar results were found for the 3-scale sensitivity composite. The final model accounted for 8% of the variance in ASQ:SE scores (*R*^2^ = 0.08), and greater household income and lower sensitivity observed during the clothing change task was associated with greater social-emotional difficulties (see Table 6).

Sensitivity analyses which were conducted by repeating the tests separately for younger and older infants found that findings were broadly consistent with those above in terms of the association with parental mental health symptoms and child development (see Supplementary Tables 5–7). However, again the strength of association between observed sensitivity and parent-reported bonding was greater for younger infants (global sensitivity ranged from *r_*s*_* = -29 to 0.37, 3-scale composite ranged from *r_*s*_* = -0.31 to -0.41) than for older infants (global sensitivity ranged from *r_*s*_* = -0.01 to -0.12, 3-scale composite *r_*s*_* = -0.04 to 0.15) (see Supplementary Table 5).

## 4 Discussion

In a clinical sample of parents and their infants, the NICHD global sensitivity scale showed construct validity to parental mental health symptoms. In contrast to predictions, this study did not find statistically significant associations between the global sensitivity scale and children’s cognitive, language or socio-emotional development, except when sensitivity was measured in the clothing change task, where lower observed sensitivity was associated with greater socio-emotional problems. The 3-scale sensitivity composite showed the same pattern of association with parental mental health symptoms and child development as the global sensitivity scale. In this study, observed sensitivity (both the global sensitivity scale and 3-scale composite) was associated with parent-reported bonding difficulties but this effect appeared to be driven by the inclusion of younger infants (under 3 months of age) in the sample. In terms of inter-rater reliability of the NICHD scales in dyads with infants under 3 months of age, good inter-rater reliability was demonstrated for the global sensitivity scale and the 3-scale composite, but poor inter-rater reliability was found for one aspect of the composite (intrusiveness). Together, it suggests that the global sensitivity scale may have advantages for use in a perinatal mental health setting where infants can be of all ages, and for purposes of improving clinical feasibility by reducing the amount of time for training and administration.

### 4.1 Reliability

In this study, that tested the use of the NICHD Scales with dyads where infants were under 3 months of age, acceptable inter-rater reliability was found for most of the parent scales, in that high levels of inter-rater agreement were achieved, but this was not the case for parental intrusiveness, dyadic mutuality and some of the infant scales. These findings are perhaps expected given the NICHD scales were originally devised for infants 3 months or older, but are in contrast to Lakes et al. (2017) who found good to excellent inter-rater reliability for almost all of the NICHD Scales in their sample of premature infants in the NICU. One reason for this may be differences in the structure of the observation task between these two studies (holding the infant in an unstructured observation versus the range of observation tasks used in this study). Consistent with what was described by Lakes et al. (2017), rating infant behavior was not always possible resulting in coders assigning a “could not code” score in some cases. The frequency of this code was less common than what was reported by Lakes et al. (2017), although the poor inter-rater reliability for the infant scales in this study suggests that coders may not have agreed about when the infant behavior was sufficiently non-observable. The most cited reason for being unable to rate infant behavior in this study was infant drowsiness which may also account in part for the poor inter-rater reliability of the intrusiveness scale for these younger infants (e.g., where a parent was trying to keep the infant awake). As the interaction progressed, this could have become more pronounced explaining the especially low agreement for intrusiveness in the final clothing change task. Finally, some of the infant scales such as positive mood require communication from the infant that is not developmentally appropriate for newborns (e.g., smiling and laughter).

### 4.2 Construct validity

As was expected, a small but significant negative association was found between parental mental health symptoms and observed parental sensitivity. The strength of this association is in line with a priori expectations and adds to existing research for the severity of depressive symptoms (Bernard et al., 2018). One might have expected this association to be of a greater magnitude in a clinical sample, however the small association is likely due to restricted variability in the range of scores, a finding that was also noted in the Bernard et al. (2018) meta-analysis examining the association between maternal depression and maternal sensitivity.

Of note is that the size of the association between sensitivity and parental mental health was smaller (and not statistically significant) when sensitivity was measured in the free-play when compared to sensitivity measured during the book sharing and clothing change task. The clothing change task elicited more infant distress in this sample, and thus may have caused more emotional dysregulation in the parent interfering with the capacity to respond sensitively (Gao et al., 2023). It is also possible that parents with more severe symptoms experienced the structured tasks (e.g., book sharing or clothing change) as more stressful due to wanting to “get it right” and that this revealed more difficulties in the interaction; an experience that has been reported in a qualitative study evaluating a video-feedback intervention in a similar sample of parents from secondary care mental health services (Barnicot et al., 2023).

Although it was predicted that observed sensitivity measured across all tasks would be associated with children’s cognitive, language and socio-emotional development, only task specific effects were found (i.e., lower sensitivity observed during the clothing change task associated with poorer socio-emotional development). This is perhaps not surprising given there is other evidence that sensitivity to distress is more predictive of socio-emotional outcomes than sensitivity to non-distress (Leerkes et al., 2009), and therefore it may be that the clothing change task presents an observation context that is particularly relevant to socio-emotional development. It would be of interest for future research to further test the validity of sensitivity observed in a clothing change task in a perinatal sample, especially given research in samples of older children to suggest that parenting observed during structured tasks may be more predictive of later externalizing problems than parenting observed in unstructured tasks (Nordahl et al., 2020).

The small strength of association between observed sensitivity in the clothing change task and socio-emotional development in this study is in line with other research that has examined parental sensitivity in relation to internalizing and externalizing problems (Cooke et al., 2022). In Cooke et al. (2022) meta-analysis they found the kind of sensitivity scale used to be a significant moderator of the association between parental sensitivity and internalizing problems (greater effects size in studies that used a sensitivity composite rather than a single sensitivity scale), where this was not the case for externalizing problems. In this study the strength of association between socio-emotional development and sensitivity measured using the global sensitivity scale (i.e., a single scale) and a sensitivity composite was comparable. However, the ASQ:SE (used in this study as a measure of socio-emotional outcomes) does not evaluate socio-emotional problems in terms of internalizing and externalizing problems in the same way as other commonly used measures in the literature and therefore it is difficult to make comparisons. It is also the case that the average age of the infants in this study is considerably younger than most studies included in Cooke et al. (2022). Thus, it is possible that the NICHD global sensitivity scale and the sensitivity composite may show different patterns of association if children’s socio-emotional outcomes are considered in terms of internalizing or externalizing problems or may do as children become older.

This study did not see any overall or task specific effects of observed sensitivity in terms of children’s cognitive or language outcomes. It may be that the cross-sectional design of this study and the average age of infants (6.5 months for the sample where ASQ data was available) meant any effects could not yet be detected for these areas of development and would become more evident in later follow up, as has been shown in longitudinal studies of children of mothers who experienced post-natal depression, particularly when poor parental mental health is chronic or recurs (Hentges et al., 2020; Murray et al., 2015; Sutter-Dallay et al., 2011).

A puzzling finding that emerged from the regression models testing the relationship between observed sensitivity and socio-emotional development was that household income was another significant predictor but in the opposite direction to what would be expected (more household income associated with greater socio-emotional problems). It may be that the use of household income as a dichotomous measure in this study influenced the usual pattern that would be expected. It could also be that increased household income is a proxy for other contextual factors within the home—such as less social support or reduced parent–child interaction due to work commitments—however this would need to be considered in future research.

In this sample, there was a small but significant association between observed sensitivity and parent-reported bonding. However, the strength of association was greater for dyads with infants under 3 months of age than for infants 3 months or older. In other studies that have examined the PBQ in relation to observed parenting, the strength of association has varied with some finding a negligible association (e.g., Nath et al., 2020 in a low-risk community sample measured at 3 months postpartum) and others findings a strong negative association between parent-reported bonding difficulties and observed parenting when controlling for other factors in a high-risk community sample measured at 6 months postpartum (e.g., Muzik et al., 2013). Findings from this study suggest that how parents perceive their bond with their baby is more strongly associated with sensitive parenting in the early postpartum period. It is possible that a greater number of factors affect both parental sensitivity and perception of the bond, as the child grows which is why this association is weaker in the older infants. However, it is also possible that something else related to infant age is moderating this association. For example, characteristics in the infant (e.g., temperament) or characteristics of the parent such as their capacity to “mentalize” (e.g., allowing the parent to remain sensitive in their interactions even if they feel less bonded), which could be avenues for future research.

### 4.3 Implications for practice and future research

This study has implications for clinical services who may wish to use the NICHD Scales as part of clinical care. It suggests that the global sensitivity scale is meaningfully (although weakly) associated with parental mental health symptom severity and children’s socio-emotional outcomes in a clinical perinatal sample and can be used reliably even amongst dyads with infants under 3 months of age for all observation tasks. The use of one scale from the NICHD system would substantially reduce time for training and administration in clinical practice. If the 3-scale sensitivity composite, is used amongst dyads with infants under 3 months of age, additional descriptors in the manual would be required to rate intrusiveness reliably and further evaluation is recommended. Considerably more refinement would be required to facilitate use of the whole NICHD system for infants this young.

Clinically, there may be value in observing parents in both structured and unstructured tasks given the overall pattern in this study that sensitivity observed in the book sharing and clothing change tasks showed stronger (although still weak) associations with other variables than when measured in the free play task. It was also the case that the global sensitivity scale and the sensitivity composite were applied reliably across the different observation tasks (ICC of at least 0.7), even with very young infants. Given the structured tasks included in this study involve simple, everyday interactions (e.g., book sharing and clothing change), they may be feasible for practitioners to use when assessing parent-infant interaction in routine clinical settings. However, how acceptable different observation tasks are to parents warrants further exploration. Future research could address how different observation tasks can be used to identify strengths and areas of need in the parent’s interaction with their infant and how the global sensitivity scale performs in response to treatment. It would be pertinent to also validate the use of this scale in a sample of fathers, partners or other caregivers to inform how PMHS might support the needs of all parents in their relationship with their infant. Future research is also needed to better understand other modifiable factors (beyond symptom severity) that are driving differences in sensitivity in a sample of parents with clinical levels of poor mental health. It is worth noting that mean levels of sensitivity reported in this sample are in line with a high-risk community sample in the UK (Sharp et al., 2024) and thus, it suggests that maternal mental health is not in and of itself a risk for low parental sensitivity (at least when observed during a brief interaction). It remains an important empirical question to determine for whom parent-infant focused interventions are required, in addition to effective treatment of parental mental health symptoms (Howard and Challacombe, 2018), and this is also true for parents accessing PMHS.

Findings from this study suggest that whilst there was a statistically significant association between parent reported bonding difficulties and observed sensitivity, this association was small (or moderate amongst dyads with younger infants), suggesting that each measure—parent self-report about bonding and direct observation of parent-infant interaction—provide unique information. Our recommendation is that services consider using both kinds of measures, alongside other clinical information, to achieve a comprehensive, multi-method assessment of the parent-infant relationship.

### 4.4 Strengths and limitations

A considerable strength of this study is the sample size of parent-infant observations in a clinical sample. Two systematic reviews of observational measures have noted that many validation studies were limited by sample sizes of fewer than 50 participants (Lotzin et al., 2015; Shone et al., 2025). It also adds to the limited literature that has tested the psychometric properties of observational measures which has been consistently identified as a research need (Lotzin et al., 2015; National Collaborating Centre for Mental Health [UK], 2015; Shone et al., 2025).

There are several limitations to consider. The nature of the sample may not be representative of all parents who access PMHS given the inclusion criteria for the trial which required a PBQ score above 12. Not all parents who access PMHS will score above this threshold, and it may exclude parents who display low sensitivity with their infant but do not perceive any problems with bonding. Another limitation is that the study was cross-sectional, and the predictive validity to later child development is believed to be a crucial psychometric property for parent-infant observation tools (Lotzin et al., 2015). Although the predictive validity of the NICHD Scales for children’s later socio-emotional outcomes has been conducted in community samples (Forrer et al., 2024a; Nordahl et al., 2020; Sharp et al., 2024), this should be a priority in terms of replication in clinical samples. The experimental design of this study meant that it was only possible to conduct cross-sectional analyses using baseline measures.

This study performed multiple tests which raises risk of Type I errors. We have focused on the strength of effects in our interpretation and did not apply stringent statistical corrections such as Bonferroni correction. Although such corrections are not considered appropriate when testing individual hypotheses (García-Pérez, 2023); Bonferroni correction could have been applied for analyses related to the different observation tasks, in which case not all *p*-values would be statistically significant, and thus these results should be interpreted with caution.

Furthermore, there are methodological considerations to consider when interpreting findings related to observed sensitivity in the different observation tasks. Firstly, the order of tasks was not counterbalanced and therefore the clothing change task was always the final task. It could be that parents or infants became more unsettled or stressed the longer the interaction went on, rather than any effects being related to the clothing change task specifically. This would be consistent with other work that has found a statistically significant reduction in sensitivity over time when a free play was followed by a teaching task (Muzik et al., 2017). In the current sample, observed sensitivity showed a statistically significant decline following the free play task, but there was no statistically significant difference in sensitivity between the book sharing and the clothing change task (see Supplementary Figure 1). It was also not possible to facilitate independent coding of each task within each dyad and therefore sensitivity observed in one task may have influenced ratings for the other tasks. Finally, because the length of each task in this study was < 5 min (which has previously found to be optimal; Sharp et al., 2024), results from this study warrant replication to see if sensitivity observed in a similar task of the same duration shows association with socio-emotional outcomes. However, the duration of the tasks was the same as procedures that have been used for other RCTs conducted in the perinatal period (Stein et al., 2018).

The measures used for child development outcomes have limitations due it to being a parent-self report and the potential for it to be affected by parental mood (Ordway, 2011). Using a parent-report alongside an independent clinical assessment of child development would have yielded a more robust assessment. An advantage to the using the ASQ in a perinatal sample is that it is suitable for use as early as 2 months of age however, because a proportion of infants in this sample were younger than 2 months of age, these dyads were not represented in analyses that tested the construct validity of the sensitivity scales to child development outcomes.

Finally, this study is limited by the characteristics of the sample which had little ethnic variability although the ethnicity profile was representative of the local area of the recruitment sites. There is a need for specific validation of measures of parental sensitivity in ethnically diverse samples in order to optimize access to interventions and reduce inequalities in child outcomes that exist in the UK (Cattan et al., 2024; Mesman et al., 2012).

## 5 Conclusion

This is the first study to test the NICHD scales in a clinical sample in England contributing to the evidence base of their psychometric properties and informing the selection of an observational measure of parent-infant interaction for use in PMHS. The global sensitivity scale and a sensitivity composite from the NICHD system demonstrated evidence of cross-sectional construct validity in a clinical sample of parents and their infants with findings that are broadly in line with those reported in community samples. The global and composite scale also demonstrated good inter-rater reliability amongst dyads with infants younger than 3 months of age, however not all the NICHD scales are suitable for use with this age group. Further psychometric research is needed in clinical samples to inform clinical care and refine treatment pathways to offer more tailored, effective support.

## Data Availability

The data analyzed in this study is subject to the following licenses/restrictions: Questionnaire data but not video recordings generated during the current study will be available as de-identified data upon request beginning 12 months and ending 5 years after the primary publication and pre-planned secondary analysis, following approval of a methodologically sound proposal and a signed data sharing agreement. Requests to access these datasets should be directed to Prof. Peter Fonagy (p.fonagy@ucl.ac.uk) and Dr. Camilla Rosan (camilla.rosan@annafreud.org).
