# Supplementary material for: A psychometric evaluation of the NICHD Parent-Infant Interaction Scales to inform clinical practice
Source: Front Psychol. 2026 Mar 3;17:1773282. doi: 10.3389/fpsyg.2026.1773282 (PMC12992259; doi:10.3389/fpsyg.2026.1773282)
Supplement: Supplementary file 1 [file Supplementary_file_1.docx]

# **Supporting Material**

**Table S1.** *Summary Description of NICHD Mother-Infant Interaction Scales (complied from Cox & Crnic, 2003)*

| **Parent Scales** | |
| --- | --- |
| Sensitivity to Distress | How the parent responds to the child’s cries, frets, or other expressions of negative affect. Considers: 1) proportion of signals responded to, 2) latency of response, 3) appropriateness of response. |
| Sensitivity to Non-Distress | How parent observes and responds to the child’s social gestures, expressions and signals. The extent to which the interaction is well-timed and paced to child’s responses and appears “in sync”. |
| Global Sensitivity | Overall sensitivity to distress and non-distress signals during the interaction. |
| Positive Regard | The degree of positive feelings expressed towards the child (verbally and nonverbally). |
| Intrusiveness | The extent to which the interaction is adult-centred rather than child-centred (e.g., the parent imposing own agenda, failing to modulate behaviour in response to child’s signals). |
| Negative Regard | The degree of negative feelings expressed towards the child (verbally or non-verbally). |
| Detachment/Disengagement | The extent to which the mother appears emotionally uninvolved or disengaged and unaware of the child’s needs for appropriate interaction to facilitate involvement with objects or people. |
| Animation | The degree of energy, excitement or interest during the interaction. |
| Stimulation of Development | The degree to which the parent tries to foster the child’s cognitive, motor or language development. |
| **Child Scales** | |
| Positive Mood | The extent to which the child is satisfied, content and pleased with the interaction. Includes positive affect and bodily enthusiasm. |
| Negative Mood | The frequency and intensity of crying, fussing, frowns or other signals of discontentment. |
| Activity | The degree of motor activity the child initiates during the interaction. |
| Sustained attention | The degree to which the child is involved in and engagement with the physical world. |
| **Dyadic Scale** | |
| Dyadic mutuality | The synchrony (e.g., intimacy and co-ordination) of interaction and the degree of shared experience. |

**Table S2.** *Length of observation by task*

|  | **Freeplay**  **(N= 272)** | **Booksharing**  **(N= 270)** | **Clothing Change**  **(N= 266)** |
| --- | --- | --- | --- |
| **Mean duration** | 2 minutes 44 seconds | 2 minutes 55 seconds | 2 minutes 27 seconds |
| **Median duration** | 2 minutes 39 seconds | 2 minutes 47 seconds | 2 minutes 18 seconds |

**Table S3.** *Descriptive Statistics for the NICHD Parent-Infant Interaction Scales for each task*

|  | **Mean**  **(SD)** | | | | **Range**  **Min, Max** | | | | **N** | | | |
| --- | --- | --- | --- | --- | --- | --- | --- | --- | --- | --- | --- | --- |
|  | **Free-Play** | **Book Sharing** | **Clothing Change** | **Total** | **Free-Play** | **Book Sharing** | **Clothing Change** | **Total** | **Free-Play** | **Book Sharing** | **Clothing Change** | **Total** |
| Global Sensitivity | 3.90  (.93) | 3.40  (1.03) | 3.37  (1.07) | 3.56  (.85) | 1, 5 | 1, 5 | 1, 5 | 1.33, 5 | 272 | 270 | 266 | 274 |
| 3-scale composite | 12.27  (2.17) | 11.04  (2.49) | 11.15  (2.48) | 11.49  (2.04) | 6, 15 | 4, 15 | 4, 15 | 6, 15 | 270 | 269 | 266 | 274 |

*Note:* †reverse scored ; higher scores indicate better interactional quality

**Table S4**. *Nonparametric inter-correlations*

|  | **1** | **2** | **3** | **4** | **5** | **6** | **7** | **8** |
| --- | --- | --- | --- | --- | --- | --- | --- | --- |
| 1. Global Sensitivity^a^ | 1.00 |  |  |  |  |  |  |  |
| 1. 3-scale sensitivity composite^a^ | .95^**^ | 1.00 |  |  |  |  |  |  |
| 1. CORE-OM total mean score | -.16^**^ | -.16^**^ | 1.00 |  |  |  |  |  |
| 1. PBQ total score | -.20^**^ | -.20^**^ | .39^**^ | 1.00 |  |  |  |  |
| 1. ASQ-3 Communication (z-score) | .06 | .02 | .10 | -.10 | 1.00 |  |  |  |
| 1. ASQ-3 Problem Solving (z-score) | .05 | .05 | -.09 | -.03 | .42^**^ | 1.00 |  |  |
| 1. ASQ-3 Personal Social (z-score) | -.003 | .02 | -.02 | -.05 | .42^**^ | .55^**^ | 1.00 |  |
| 1. ASQ: SE (z-score) | -.10 | -.13 | .12 | .23^**^ | -.19^**^ | -.08 | -.09 | 1.00 |

^a^ measured across all tasks

^*^*p*≤.05

^**^*p*≤.01

**Table S5.** *Age-specific non-parametric correlations between observed sensitivity and maternal mental health and bonding difficulties*

|  | **YOUNGER INFANTS**  **(Under 12 weeks)**  **N=75^†^** | **OLDER INFANTS**  **(12 weeks or older)**  **N=195^a^** |
| --- | --- | --- |
| **Total sensitivity (mean across all tasks)** | | |
|  | **CORE-OM** | **CORE-OM** |
| Global Sensitivity | *r_s_*= -.21, *p*=.07 | *r_s_* =-.17, *p*=.02 |
| 3-scale composite | *r_s_* =-.21, *p*=.08 | *r_s_* =-.15, *p*=.04 |
|  | **PBQ** | **PBQ** |
| Global Sensitivity | *r_s_* = -.41, *p*<.001 | *r_s_* =-.10, *p*=.17 |
| 3-scale composite | *r_s_* =-.40, *p*<.001 | *r_s_* =-.11, *p=*.12 |
| **Free play** | | |
|  | **CORE-OM** | **CORE-OM** |
| Global Sensitivity | *r_s_* =-.08, *p*=.51 | *r_s_* =-.05, *p*=.49 |
| 3-scale composite | *r_s_* =-.20, *p*=.09 | *r_s_* =-.06, *p*=.43 |
|  | **PBQ** | **PBQ** |
| Global Sensitivity | *r_s_* = -.29, *p*=.01 | r=-.01, *p*=.87 |
| 3-scale composite | *r_s_* =-.34, *p*=.003 | r=-.04, *p*=.61 |
| **Booksharing** | | |
|  | **CORE-OM** | **CORE-OM** |
| Global Sensitivity | *r_s_* =-.20, p=.10 | *r_s_* = -.21, *p*=.004 |
| 3-scale composite | *r_s_* =.-14, p=.24 | *r_s_* = -.15, *p*=.03 |
|  | **PBQ** |  |
| Global Sensitivity | *r_s_* =-.37, p=.001 | *r_s_* = -.12, *p=*.10 |
| 3-scale composite | *r_s_* =-.31, p=.008 | *r_s_* =-.07, *p*=.33 |
| **Clothing Change** | | |
|  | **CORE-OM** | **CORE-OM** |
| Global Sensitivity | *r_s_* =-.25, *p*=.04 | *r_s_* =-.16, *p*=.03 |
| 3-scale composite | *r_s_* =-.24*, p*=.05 | *r_s_* =-.18, *p*=.01 |
|  | **PBQ** | **PBQ** |
| Global Sensitivity | *r_s_* =-.37, p=.002 | *r_s_* =-.09, *p*=.21 |
| 3-scale composite | *r_s_* =-.41, p<.001 | *r_s_* =-.15, *p*=.05 |

^†^ *except for book sharing=73, clothing change=70*

*^a^ N for PBQ=194, free play= 193, book sharing and clothing change=192*

**Table S6.** *Age specific* *results for linear regression of child development outcomes on total observed sensitivity (older infants only, N=177)*

|  | **ASQ-3** | | | | | | **ASQ:SE** | |
| --- | --- | --- | --- | --- | --- | --- | --- | --- |
|  | **Communication** | | **Problem Solving** | | **Personal Social** | | **Socio-emotional** | |
| **Predictor** | **B**  **(BCa 95% CI)** | ***p*** | **B**  **(BCa 95% CI)** | ***p*** | **B**  **(BCa 95% CI)** | ***p*** | **B**  **(BCa 95% CI)** | ***p*** |
| Global sensitivity | .06 (-.13, .24) | .56 | .004 (-.17, .18) | .97 | -.08 (-.26, .10) | .37 | -.07 (-.24, .11) | .46 |
| 3-scale composite | -.003 (-.08, .08) | .93 | .000 (-.08, .08) | .995 | -.03 (-.10, .04) | .40 | -.04 (-.11, .04) | .33 |

*Note:* BCa CI: Bias corrected accelerated 95% bootstrap confidence interval (2000 resampling iterations); ASQ-3 = Ages and Stages Questionnaire- Third Edition, lower scores indicate poorer development; ASQ:SE = Ages and Stages Questionnaire: Socio Emotional, higher scores indicate greater socio-emotional problems

**Table S7.** *Age specific results for linear regression of child development outcomes on observed sensitivity by task (older infants only)*

|  | **ASQ-3 (N=172^†^)** | | | | | | **ASQ:SE (N=171^††^)** | |
| --- | --- | --- | --- | --- | --- | --- | --- | --- |
|  | **Communication** | | **Problem Solving** | | **Personal Social** | | **Socio-emotional** | |
| **Predictor** | **B**  **(BCa 95% CI)** | ***p*** | **B**  **(BCa 95% CI)** | ***p*** | **B**  **(BCa 95% CI)** | ***p*** | **B**  **(BCa 95% CI)** | ***p*** |
| Global sensitivity | |  |  |  |  |  |  | |
| Free play | .06 (-.11, .23) | .53 | .05 (-.13, .23) | .41 | -.08 (-.24, .08) | .34 | .04 (-.12, .23) | .67 |
| Book sharing | -.08 (-.28, .11) | .42 | -.08 (-.24, .07) | .41 | -.09 (-.28, .11) | .36 | .09 (-.08, .26) | .32 |
| Clothing Change | .06 (-.11, .23) | .50 | .05 (-.13, .22) | .58 | .07 (-.11, .24) | .43 | -.17 (-.33, -.03) | .03^a^ |
| 3-scale composite | | |  |  |  |  |  | |
| Free play | .02 (-.06, .10) | .61 | .03 (-.05, .11) | .43 | -.02 (-.097, .06) | .58 | .002 (-.07, .08) | .95 |
| Book sharing | -.04 (-.11, .04) | .35 | -.01 (-.09, .05) | .69 | -.04 (-.11, .05) | .37 | .05 (-.03, .14) | .18 |
| Clothing Change | .01 (-.07, .09) | .82 | -.003 (-.08, .07) | .95 | .02 (-.06, .09) | .62 | -.08 (-.15, -.01) | .03^b^ |

*Note:* BCa CI: Bias corrected accelerated 95% bootstrap confidence interval (2000 resampling iterations); ASQ-3 = Ages and Stages Questionnaire- Third Edition, lower scores indicates poorer development; ASQ:SE = Ages and Stages Questionnaire: Socio Emotional, higher scores indicate greater socio-emotional problems; ^†^169 for 3-scale composite; ^††^168 for 3-scale composite; **^a^** predictor remains significant when maternal age, education, household income, maternal mental health symptoms are entered into the model B= -.22 (-.39, -.07), p=.01; ^b^ predictor remains significant when maternal age, education, household income, maternal mental health symptoms are entered into the model B= -.08 (-.16, -.01), p=.02

**Figure S1.** *Mean Rank Sensitivity Over Time*

Results from a Friedman test show that there was a statistically significant difference in global sensitivity by task, χ^2^(2) = 79.23, *p <.*001. Post hoc analysis with Wilcoxon signed-rank tests indicate that there were no significant difference between global sensitivity during the book sharing and clothing change task (Z = -.64*, p* = 0.52) but there was a statistically significant reduction in global sensitivity observed between the free play and book sharing task (Z = -7.92, *p* <.001), and free play and clothing change task (Z= -7.648, *p*<.001).
